# Rejection of murine mammary tumours in BALB/c mice.

**DOI:** 10.1038/bjc.1979.13

**Published:** 1979-01

**Authors:** S. Robidoux, A. Kajdos, M. Tomana, C. W. Niedermeier


*Br. J. Cancer* (1979), **39**, 99

***Short Communication***

### REJECTION OF MURINE MAMMARY TUMOURS IN BALB/c MICE

S. ROBIDOUX, A. KAJDOS, M. TOMANA* AND C. W. NIEDERMEIER

*From the Division of Clinical Immunology and Rheumatology and the Comprehensive Cancer Center, University of Alabama in Birmingham, Alabama* 35294, *USA*

Received 29 September 1978 Accepted 10 October 1978

THE PRESENCE of circulating antibodies to murine mammary tumour virus (MuMTV) related antigens in mice as well as in humans has been reported by a number of investigators (Muller *et al.*, 1976; Priori *et al.*, 1972; Hoshino & Dmochowski 1973). Tumour growth, according to a hypotheses of Nordquist *et al.* (1977) is facilitated by antibody-induced redistribution and shedding of mammary-tumour antigens into the circulatory system. The antigen-denuded cells are not then recognized by effector agents. Efforts to inhibit mammary-tumour growth and reduce its incidence in susceptible strains of mice by immunization with MuMTV were not successful (Charney *et al.*, 1976). Sarkar & Moore (1978), however, have recently shown that C57BL mice that were immunized with purified formalin-inactivated MuMTV showed increased resistance to exogenous MuMTV infection, and to subsequent development of mammary tumours. They concluded that successful immunization required the use of relatively high doses of killed virus.

The recorded incidence of spontaneous-tumour rejection by inbred mice is very rare. Hewitt *et al.* (1976) found that, in ~ 20,000 transplantations of different tumours in WHT/Ht and CBA/Ht lines of mice, only 2 cases of tumour rejection occurred.

The purpose of this study was to explain the repeated rejection of mouse mammary-tumour implants by 2 mice in our laboratory, and to determine whether these mice produced antibody reactive against human breast-tumour tissue.

In the course of 2 years, murine mammary tumours derived from BALB/cf C3H mice were implanted into 290 mice. Tumours were allowed to grow to a size of about 1 $cm^3$ before s.c. transplantation of 2 pieces of tumour tissue (1 $mm^3$) in the lateral abdominal area of recipient mice. The tumours were propagated in the animals for 10 generations. Only 2 mice, one in the 4th and the other in the 7th generation, rejected the tumours. Three separate attempts to implant tumours were made in each mouse, without success. At each attempt the same tumour was successfully implanted into several other mice.

Sera obtained by retro-orbital sinus bleeding from the mice that rejected the tumour, 8 healthy mice, and 3 tumour-bearing mice from the same colony, were absorbed with lyophilized human benign breast-tumour tissue and examined for the presence of antibodies to antigens in human breast carcinomas. Sera from the 2 mice that rejected the tumors reacted positively when tested by indirect immunofluorescence for reactivity against frozen sections of human breast adenocarcinomas. FITC-labelled anti-mouse IgG serum from Meloy Laboratories was used as the secondary antibody in the indirect immunofluorescence assay. Sera from all other mice were negative. All sera were

* Author to whom reprint requests should be addressed.

negative when tested against sections of human benign breast tumours. Positive immunofluorescence was also seen when sections of human breast adenocarcinomas were examined for reactivity with rabbit anti-MuMTV serum. The results are shown in the Table.

TABLE.—*Reactivity of mouse sera with human breast-tumour sections*

| Origin of serum | No of animals | Benign | Carcinoma |
|---|---|---|---|
| Mice rejecting tumour | 2 | — | + |
| Healthy mice | 8 | — | — |
| Tumour-bearing mice | 3 | — | — |
| Rabbit anti-MuMTV | | — | + |

The results of this investigation indicate that sera from the mice that rejected the mammary tumours contained antibodies directed against antigens in human breast-carcinoma tissue. The immunological reactivity of sera from these mice was similar to the reactivity of rabbit anti-MuMTV sera when tested against sections of human breast tumour. The sera of control and tumour-bearing mice did not possess this reactivity.

It has been previously shown (Muller *et al.*, 1976; Priori *et al.*, 1972; Hoshino & Dmochowski, 1973; Tomana *et al.*, 1979) that an immunological relationship exists between mouse and human breast cancer. Although the existence of a human breast-tumour virus is still questionable, components were detected in human breast-carcinoma patients that are antigenically related to a component of MuMTV. Although our results suggest that the mice rejecting the tumour had natural or spontaneously acquired antibodies to MuMTV, it is possible that the piece of tumour used for the first implantation consisted of non-viable tissue which resulted in immunization of the mouse. Rejection of the 2nd and 3rd implants that were done at about one-month intervals could thus be explained. This explanation is consistent with previous findings of Sarkar & Moore (1978) who demonstrated successful immunization of mice with formalin-treated MuMTV. The results of this investigation support the hypothesis that there is an aetiologic relationship between mouse and human breast carcinomas.

This investigation was supported by grants CA-19918, CA-19933 and contract NO1-CB7092, awarded by the National Cancer Institute, DHEW.

REFERENCES

CHARNEY, J., HOLBEN, J. A., CODY, C. M. & MOORE, D. H. (1976) Further immunization studies with mammary tumor virus. *Cancer Res.*, **36**, 777.

HEWITT, H. B., BLAKE, E. R. & WALSEN, A. S. (1976) A critique of the evidence for active host defense against cancer, based on personal studies of 27 murine tumours of spontaneous origin. *Br. J. Cancer*, **33**, 241.

HOSHINO, M. & DMOCHOWSKI, L. (1973) Electron microscope study of antigens in cells of mouse mammary tumor cell lines by perioxidase-labeled antibodies in sera of mammary tumor-bearing mice and of patients with breast cancer. *Cancer Res.*, **33**, 2551.

MULLER, M., ZOTTER, S. & KEMMER, C. (1976) Specificity of human antibodies to intracytoplasmic type-A particles of the murine mammary tumor virus. *J. Natl Cancer Inst.*, **56**, 295.

NORDQUIST, R. E., ANGLIN, J. H. & LERNER, M. P. (1977) Antibody-induced antigen redistribution and shedding from human breast cancer cells. *Science*, **197**, 355.

PRIORI, S. E., ANDERSON, D. E., WILLIAMS, W. C. & DMOCHOWSKI, L. (1972) Immunological studies on human breast carcinoma and mouse mammary tumors. *J. Natl Cancer Inst.*, **48**, 1131.

SARKAR, N. H. & MOORE, D. H. (1978) Immunization of mice against murine mammary tumor virus infection and mammary tumor development. *Cancer Res.*, **38**, 1468.

TOMANA, M., YAGI, M. J., NIEDERMEIER, W., ROBIDOUX, S. & MESTECKY, J. (1979) Specificity of antibodies in human sera to MMTV-related antigens of human breast carcinoma tissues. *Clin. Immunol. Immunopathol.* (In Press).


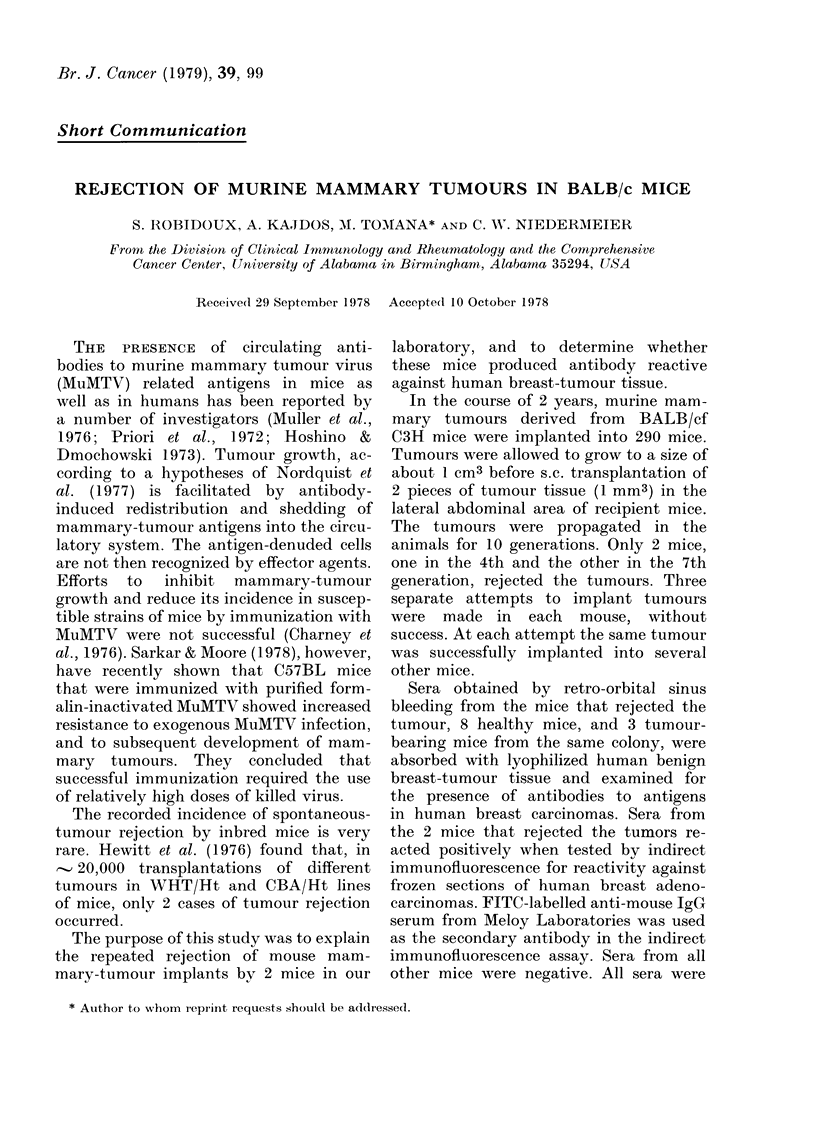

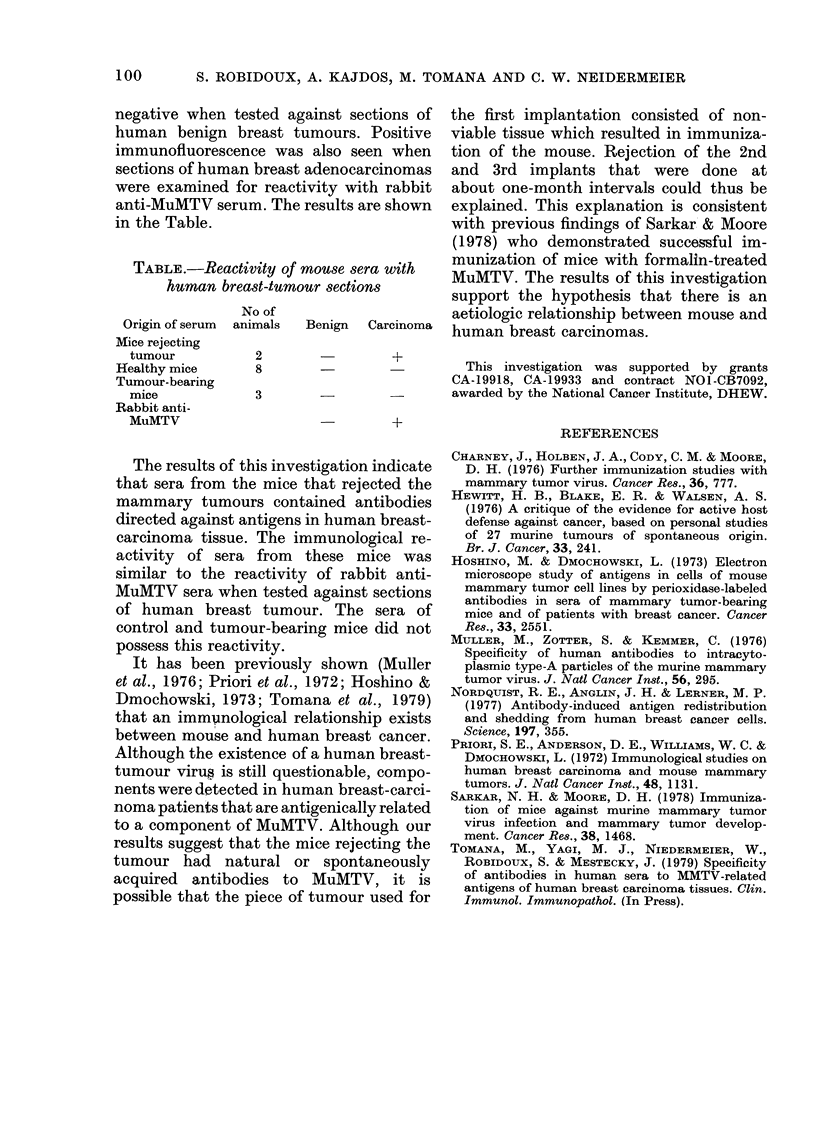


## References

[OCR_00172] Charney J., Holben J. A., Cody C. M., Moore D. H. (1976). Further immunization studies with mammary tumor virus.. Cancer Res.

[OCR_00177] Hewitt H. B., Blake E. R., Walder A. S. (1976). A critique of the evidence for active host defence against cancer, based on personal studies of 27 murine tumours of spontaneous origin.. Br J Cancer.

[OCR_00184] Hoshino M., Dmochowski L. (1973). Electron microscope study of antigens in cells of mouse mammary tumor cell lines by peroxidase-labeled antibodies in sera of mammary tumor-bearing mice and of patients with breast cancer.. Cancer Res.

[OCR_00192] Müller M., Zotter S., Kemmer C. (1976). Specificity of human antibodies to intracytoplasmic type-A particles of the murine mammary tumor virus.. J Natl Cancer Inst.

[OCR_00204] Priori E. S., Anderson D. E., Williams W. C., Dmochowski L. (1972). Immunological studies on human breast carcinoma and mouse mammary tumors.. J Natl Cancer Inst.

[OCR_00210] Sarkar N. H., Moore D. H. (1978). Immunization of mice against murine mammary tumor virus infection and mammary tumor development.. Cancer Res.

